# High proportion of hepatitis B virus-infected patients at a mild stage of disease progression: a cross-sectional study in a single reference laboratory in Cameroon

**DOI:** 10.11604/pamj.2025.51.13.45939

**Published:** 2025-05-14

**Authors:** Pretty Rosereine Mbouyap, Laure Ngono, Chavely Gwladys Monamele, Jeanne Manga, Fréderic Lissock, Annie Epote, Suzanne Belinga, Richard Njouom

**Affiliations:** 1 Centre Pasteur, Yaounde, Cameroon,; 2 Faculty of Medicine and Biomedical Sciences, University of Yaounde I, Yaounde, Cameroon

**Keywords:** Chronic HBV infection, HBV viral load, ALT, AST, HbeAg

## Abstract

**Introduction:**

hepatitis B causes significant suffering owing to acute and chronic infection leading to complications such as cirrhosis and hepatocellular carcinoma. Chronic Hepatitis B infection can be identified by the simultaneous use of virological, serological and biochemical markers involved in monitoring the progression of the disease. In Africa, only 1% of people infected are screened, and few benefit from antiviral treatment. This study aimed to determine the relationship between serological, biochemical and virological markers involved in the follow-up of patients with chronic viral hepatitis B virus infection at the Centre Pasteur du Cameroun.

**Methods:**

we conducted a cross-sectional study of patients' electronic records registered in the Laboratory Information System of the Centre Pasteur du Cameroun from 2011 to 2021. HBs antigen-positive patients with viral load, HBe antigen and liver transaminases assays were included. Chronic HBV infection phases were classified in 5 phases according to EASL which are: Phase 1: HBeAg-positive chronic HBV infection, Phase 2: HBeAg-positive chronic hepatitis B, Phase 3: HBeAg-negative chronic HBV infection, Phase 4: HBeAg-negative chronic hepatitis B, Phase 5: HBsAg-negative phase.

**Results:**

we registered 1652 patients, 43.9% females and 56.1% males. The median age of women and men was 31 [>25;38] years and 33 [6;40] years respectively. The transaminase levels were normal in the most patients (65.5% for ALT and 76.2% for AST), low Hepatitis B viral load (<2000 IU/mL) in 68.5% of cases and 90.1% were negative for HBe antigen. Regarding viral Hepatitis B stage, 3.4%, 6.5% and 28.1% were in stage 1,2 and 4 respectively. Most (62.0%) fell in stage 3 representing the mild HBeAg-negative chronic HBV infection.

**Conclusion:**

our results show that a high proportion of HBV-infected patients referred at Centre Pasteur du Cameroun are at a mild stage of disease progression.

## Introduction

Chronic hepatitis B (CHB), defined as persistence of hepatitis B surface antigen (HBsAg) for six months or longer, is a major public health problem. Worldwide, there are an estimated 296 million chronically infected persons, and nearly 650,000 Hepatitis B Virus (HBV)-related deaths are recorded every year. Cirrhosis and hepatocellular carcinoma are the main complications. Among people with a chronic infection, between 20 and 30% could develop complications [1].

The Western Pacific region and Africa are home to the majority of infected people, where almost 68% of HBV-related deaths are recorded. [2]. However, in Africa, only 1% of people infected with HBV are screened, and of these, very few benefit from antiviral treatment [2]. In Cameroon, the prevalence of HBV-infected patients is 11.2%, which classifies it as a high-prevalence area [3]. This is why a global strategy to combat viral hepatitis has been drawn up by the World Health Organization (WHO), intending to eliminate viral hepatitis as a health problem by 2030 [4]. WHO launched a global program against hepatitis B and C infections, whose aim by 2030 is to reduce the number of new cases of viral infections by 90%, reduce the number of hepatitis related deaths by 65%, and treat 80% of viral hepatitis infections. The main targeted interventions are vaccination of infants, screening and treatment of persons at risk [5].

The initiation of treatment for an HBV-infected patient depends on certain eligibility criteria defined by learned societies taking into account the different phases of the evolution of liver disease. Chronic HBV infection has different phases, which can be identified by the simultaneous use of some virological, serological and biochemical markers involved in monitoring the progression of the disease [5]. The surveillance of markers is essential for determining the different stages of the disease, adopting appropriate treatment and making the right decisions. [6]. These phases are not necessarily successive or sequential. They are defined by a new classification from the European Association for the Study of the Liver (EASL). The natural history of HBV infection can be divided into 5 phases based on the presence or absence of hepatitis B e antigen (HBeAg), the level of viral replication, the level of alanine aminotransferase (ALT) and the presence of histological lesions of inflammation or fibrosis in the liver [2]. There are five stages of HBV infection namely: (I) HBeAg-positive chronic infection, (II) HBeAg-positive chronic hepatitis, (III) HBeAg-negative chronic infection, (IV) HBeAg-negative chronic hepatitis and (V) HBsAg-negative phase. This classification is mainly based on the description of the two characteristics of chronicity, namely infection and hepatitis [7]. antiviral treatment against HBV aims to prevent, halt or reverse the progression of liver disease towards cirrhosis and liver cancer [1,7].

Patients with advanced life-threatening liver disease such as acute liver failure and cirrhosis should be treated. Individuals with minimal fibrosis and a low risk of CHB progression are not usually considered for treatment, but these individuals should be identified and followed over time [1]. It will be necessary to classify HBV-infected patients to identify patients who require treatment. This study aimed to determine the relationship between serological, biochemical, and virological markers involved in the follow-up of patients with CHB infection at *Centre Pasteur du Cameroun (CPC)*.

## Methods

**Study design and setting:** this was an analytical cross-sectional study of the electronic records of patients with chronic HBV infection, recorded in the Laboratory Information System (LIS) of the *CPC* over ten years from January 2011 to December 2021.

**Study site:** *CPC* is a technical establishment of the Ministry of Public Health of Cameroon and a reference laboratory for diagnosing and monitoring various infections in Cameroon, including HBV. The medical analysis laboratories (LAM) of the *CPC* have an accredited quality management system according to the international standard ISO 15189 (TUNAC) for hepatitis B diagnostic markers: hepatitis B surface antigen (HBsAg), anti-HBs antibody, anti-HBc antibody and HBV viral load (HBV VL). As such, this facility receives patients from across the country for routine screening and monitoring.

**Enrolment of study participants:** we retrieved from the *CPC* LIS the results of ALT, HBeAg serology, and HBV VL of HBV-infected patients (HBsAg-positive) aged over 18 years. Only patients who had carried out all three analyses performed in the biochemistry, serology and virology between January 2011 and December 2021 were included in this study.

**Procedure for ALT measurement:** ALT was measured photometrically by measuring the decrease in absorbance using the Roche/Hitachi, Cobas c 501, according to the manufacturer's instructions. Normal values for ALT were ≤40 IU/L irrespective of sex.

**Detection of HBsAg and HBeAg:** these serological analyses were performed respectively using the ARCHITECT HBsAg qualitative II assay (Abbott i1000sr) and the ARCHITECT HBeAg assay (Abbott i1000sr); for the qualitative detection of HBsAg and HBeAg in serum and plasma, respectively using a chemiluminescent microparticle immunoassay (CMIA), according to the manufacturer's instructions. Positive results for HBsAg were confirmed using the ARCHITECT HBsAg qualitative II confirmatory (Abbott i1000sr).

**HBV Viral Load determination:** the automated Abbott Real Time HBV m2000 system was used to extract and quantify HBV DNA levels. Abbott RealTime HBV assay is an in vitro polymerase chain reaction (PCR) assay for use with the Abbott m2000 System DNA reagents and with the Abbott m2000sp and m2000rt instruments for the quantitation of Hepatitis B Virus (HBV) DNA in human serum or plasma (EDTA) from chronically HBV-infected individuals. The detection limit was 10 IU/mL.

**Classification into the different phases of chronic HBV infection:** chronic HBV infection phases were classified based on EASL [4].

***Phase 1:*** HBeAg-positive chronic HBV infection, previously termed “immune tolerant” phase; characterised by the presence of serum HBeAg (>10^7^IU/mL, very high levels of HBV DNA and ALT persistently (≤40 IU/L).

***Phase 2:*** HBeAg-positive chronic hepatitis B is characterised by the presence of serum HBeAg, high levels of HBV DNA (10 4-10^7^), elevated ALT (>40 IU/L).

***Phase 3:*** HBeAg-negative chronic HBV infection, previously termed 'inactive carrier' phase or “immune control phase”, is characterised by undetectable or low (<2,000 IU/mL) HBV DNA levels and normal ALT (≤40 IU/L).

***Phase 4:*** HBeAg-negative chronic hepatitis B is characterised by the lack of serum HBeAg, persistent or fluctuating moderate to high levels of serum HBV DNA (often lower than in HBeAg-positive patients), as well as fluctuating or persistently elevated ALT values (>40 IU/L).

***Phase 5:*** HBsAg-negative phase is characterised by negative serum HBsAg and positive antibodies to HBcAg (anti-HBc), with or without detectable antibodies to HBsAg (anti-HBs). This phase is also known as “occult HBV infection”.

**Statistical analysis:** the collected data were revised, coded and entered into IBM Statistical Package for the Social Sciences (SPSS®), version 22 (SPSS Inc., IBM Corp., Armonk, NY, USA). Quantitative data are presented as means, standard deviations (SD) and ranges, whilst qualitative data are presented as numbers and percentages. Comparisons between two groups were done using the Wilcoxon-Mann-Whitney test for non-parametric continuous variables and Student's t-test for parametric quantitative variables, whilst qualitative data were compared using the chi-squared test or Fisher's exact test when the expected count in any cell was found to be <5. The confidence interval (CI) was set to 95% and the accepted margin of error was set to 5%. The p-value was considered statistically significant at p≤0.05.

**Ethical considerations:** data set had been stripped of all possible identifiers and coded before analysis. No additional laboratory tests were performed other than those requested by the clinician, and there were no supplementary data were collected. As such, no ethical approval was required.

## Results

**Figure 1:**
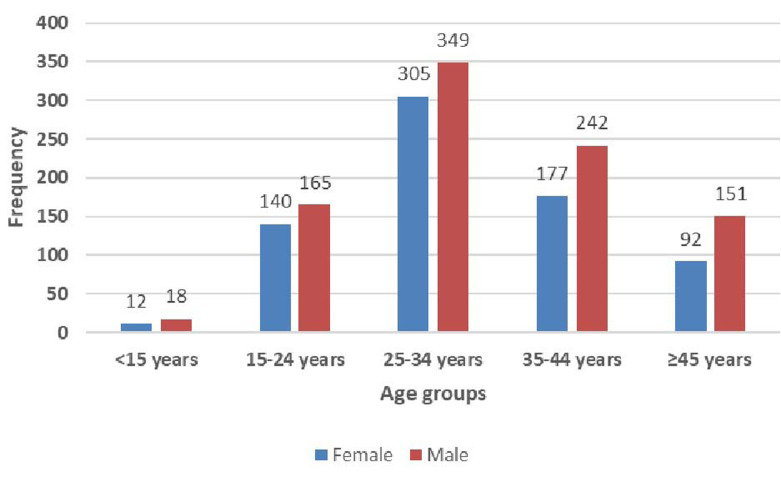
age and sex distribution of the study population

**Demographic characteristics of study population:** a total of 1652 patients with all three results for HBV VL, HBeAg serology and ALT levels were extracted. This study population was 726 women (43.9%) and 926 men (56.1%). The age distribution was similar in both the male and the female population. The median age of females was 31 [25-38] years, and that of males was 33 [26-40] years. The most represented age groups were the young adult population aged 25-34 and 35-44 years. The age and sex distribution are shown in Figure 1.

Regarding biological parameters, ALT levels ranged from 5 to 1962 IU/L with a mean value of 55.8±3.2 IU/L and a median of 33 [24;49] IU/L. AST ranged from 9 to 4645 IU/L with a mean of 45.4±3.3 IU/L and a median of 30 [23;40] IU/L. HBV VL ranged from below 10IU/mL to 10^9^ IU/mL with a mean of 1.6x10^7^ ±2.5x10^6^ IU/mL and a median of 502.0 [77;3518] IU/mL. Mean and median values of all biological parameters were higher in males compared to females as detailed in Table 1.

**Table 1:** description of study participants demographic and biological characteristics

Characteristics	Details	Total N=1652	Male N=926	Female N=726
**Age**	Range	5–76	5–75	8–76
	Mean ± SD	33.4±0.3	33.9±0.4	32.7±0.4
	Median [Q1; Q3]	32 [26; 39]	33 [26; 40]	31[25; 38]
**ALT (IU/L)**	Range	5–1962	9–1962	5–1901
	Mean ± SD	55.8±3.2	61.5±3.8	48.6±5.5
	Median [Q1; Q3]	33 [24; 49]	39 [29; 56]	28 [20; 36]
**AST (IU/L)**	Range	9–4645	13–1552	9–4645
	Mean ± SD	45.4±3.3	46.7±2.3	43.8±6.9
	Median [Q1;Q3]	30 [23; 40]	34 [26; 44]	26 [21; 34]
**HBVVL (IU/mL)**	Range	<10–10^9^	<10–10^9^	<10–10^9^
	Mean ± SD	1.6 x 10^7^±2.5 x10^6^	2.0 x 10^7^±3.7 x 10^6^	1.0 x 10^7^±3.2 x 10^6^
	Median [Q1; Q3]	502 [77; 3518]	743.5 [104; 4825]	309.5 [59; 2093]
**HBVVL (Log IU)**	Range	0.7–9.0	0.7–9.0	0.7–9.0
	Mean ± SD	2.9±0.04	3.2±0.1	2.7±0.1
	**Median [Q1; Q3]**	**2.7 [1.9; 3.6]**	**2.9 [2.0; 3.7]**	**2.5 [1.8; 3.3]**

Most participants had normal values of liver transaminases (n= 1080,65.5%) for ALT and (n=1255, 76.2%) for AST, low HBV VL below 2000 IU/mL at (n= 1131,68.5%) and (n=1489,90.1%) were HBeAg negative. Regarding HBV stage, most participants fell in stage 3 (n=1025, 62.0%), representing the relatively mild HBeAg-negative chronic HBV infection. All biological parameters, including ALT, AST, HBV VL and HBeAg were significantly higher in males compared to females (Table 2). Also, HBV stage 2 and 4, indicative of chronic hepatitis were significantly higher in males compared to females (Table 2). Concerning the association between biological parameters and two categories of age groups, no statistically significant association was found with the biochemical parameters (Table 2). Higher HBV VL and HBeAg positives were noted in the ≤30 years age group (Table 2). Also, HBV stage 2 (HBeAg positive chronic hepatitis) was significantly higher in the ≤30 years age group compared to the >30 years (Table 2).

**Table 2:** comparison of biological parameters by sex and age

Characteristics		Total N=1652	Male N (%)	Female N (%)	P-value	≤ 30 years N (%)	>30 years N (%)	P-value
ALT	Normal	1080 (65.5)	488 (52.9)	592 (81.5)	<0.001	468 (64.3)	611 (66.4)	0.367≠
	High	569 (34.5)	435 (47.1)	134 (18.5)		260 (35.7)	309 (33.6)	
AST	Normal	1255 (76.2)	629 (68.1)	626 (86.3)	<0.001	538 (74.0)	716 (77.8)	0.071≠
	High	393 (23.8)	294 (31.9)	99 (13.7)		189 (26.0)	204 (22.2)	
HBVVL	<2000	1131 (68.5)	589 (63.6)	542 (74.7)	<0.001	589 (64.4)	542 (71.8)	<0.001≠
	2000-20,000-	295 (17.9)	186 (20.1)	109 (15.0)		186 (17.9)	109 (17.8)	
	>20,000	226 (13.6)	151 (16.3)	75 (10.3)		151 (17.7)	75 (10.4)	
HBeAg	Negative	1489 (90.1%)	809 (87.4)	680 (93.7)	<0.001	617 (84.5)	872 (94.7)	<0.001≠
	Positive	163 (9.9%)	117 (12.6)	46 (6.3)		113 (15.5)	49 (5.3)	
HBV stage	Stage 1	56 (3.4%)	36 (3.9)	20 (2.8)	<0.001	41 (5.6)	14 (1.5)	<0.001≠
	Stage 2	107 (6.5%)	81 (8.7)	26 (3.6)		72 (9.9)	35 (3.8)	
	Stage 3	1025 (62.0%)	453 (48.9)	572 (78.8)		427 (58.5)	598 (64.9)	
	Stage 4	464 (28.1%)	356 (38.4)	108 (14.9)		190 (26.0)	274 (29.8)	

≠Student test

**Virological, serological and biochemical characteristics of the study population:** HBV VL <2000 IU/mL was detected in 1131/1652 (68.4%) participants. Two hundred and ninety-five participants (17.8%) had HBV VL between 2000 and 20,000 IU/mL while 226/1652 (13.6%) participants had values >20,000 IU/mL. There was a significantly higher proportion of HBeAg-positive patients with viral loads >20,000 IU/mL (n= 116, 71.2%) compared to HBeAg-negative patients (n=110, 7.4%) with an odds ratio of 30.9 (Table 3). Also, a high proportion of patients with HBV VL >20,000 IU/mL had significantly higher liver transaminase levels (n=138; 24.3% ALT and n=111; 28.2% AST) compared to their opposite counterparts with HBV VL ≤20,000 IU/mL (n=88; 8.1% ALT and n=115; 9.2% AST; P<0.001).

**Table 3:** HBV VL compared with demographic, immunological and biochemical characteristics

Characteristics		HBVVL (IU/mL)		OR	95% CI	P-value
		≤20,000 N (%)	>20,000 N (%)			
ALT	Normal	992 (91.9)	88 (8.1)	3.6	2.7 – 4.8	<0.001
	High	431 (75.7)	138 (24.3)			
AST	Normal	1140 (90.8)	115 (9.2)	3.9	2.9 – 5.2	<0.001
	High	282 (71.8)	111 (28.2)			
HBeAg	Negative	1379 (92.6)	110 (7.4)	30.9	20.9 –45.7	<0.001
	Positive	47 (28.8)	116 (71.2)			

CI: Confidence interval

## Discussion

This study aimed to determine the relationship between serological, biochemical, and virological markers involved in the follow-up of patients with CHB infection at *CPC*. We reported the virological, serological and biochemical characteristics of chronically HBV-infected patients at *CPC* to identify the association between socio-demographic and biological parameters and to determine the proportion of patients in different stages of chronic HBV infection. More than half of the participants were men, and this group had higher HBV VL and ALT levels and a higher prevalence of HBeAg-positive serology. These findings are similar to studies conducted by Tufon *et al*. and Meriki *et al*. in Cameroon in 2017 and 2021, in which men were the highest represented population at 64.3% and 55.7%, respectively [6,8]. Liver complications were more common in men than in women in some studies, and this was not related to environmental or behavioural factors [9,10]. Chronic liver disease may be influenced by the action of estrogens, which may have a protective and defensive role for liver cells [11,12]. There is an apolipoprotein (A-I) found only in male hepatocytes. This was discovered by the Chinese and could predispose to HBV infection and associated complications [13]. Estrogens produced in women can slow the progression of liver disease and limit complications [6].

In the study carried out by Esmaeelzadeh *et al*. in Iran, almost 59% of participants had transaminases above 40IU/ml and mean HBV-DNA level was 7.7±1.5×10^5^ IU/ml, ranging from zero to 14 10^7^ IU/ml [14]. This indicates a level of viral replication associated with hepatic cytolysis in a majority of cases. However, in our study, a low level of viral replication and a low level of hepatic cytolysis were observed. Regarding HBV stage, 62.0% of participants fell in phase 3. A similar result was found by Tufon *et al*. where almost 76% of participants were in phase 3 (immunocontrol) and only 18.4% of the participants fell in phase 2, while 4 were considered as the category of individuals who should be considered for treatment [6]. This is because we used the same HBV DNA viral load thresholds to classify patients into the HBeAg-negative chronic HBV infection phase.

The correlation between the level of viral load and transaminases has often been investigated in several studies. Some studies have shown a correlation between viral load and ALT [14-21]. These results are in line with those found in our study. In this way, the level of viral replication can be revealed by the ALT level. This can be an element of monitoring in our context of countries with limited resources, where molecular biology platforms are not always available.

This study also shows that many HBV-infected people are HBeAg negative and have low viral loads. Since HBV DNA levels, ALT levels, and HBeAg status are among the most important determinants of the risk of progression to cirrhosis, our participants are likely to have minimal or no liver damage [6]. The patients with HBeAg-negative CHB in this study tended to be older than those with HBeAg-positive CHB. Chu *et al*. 2007 reported that persistent HBeAg seropositivity beyond 40 years of age is relatively uncommon and is associated with a higher risk of cirrhosis and HCC [22].

There was a significantly higher proportion of HBeAg-positive patients with viral loads >20,000IU/ml compared to HBeAg-negative patients (p<0.001). A study has reported that HBeAg-positive CHB patients tend to have higher viral loads than HBeAg-negative patients [23]. Fouad *et al*. in Egypt in 2020 showed that a significant proportion (87.5%) of HBeAg-positive patients had HBV DNA levels greater than 2x10^4^ IU/mL, but this was not significantly higher than HBV DNA levels in HBeAg-negative patients (68%) [23]. Additionally, 7.4% of our study population had high HBV VL >20,000 IU/mL and were HBeAg negative. Since certain preC/C mutations have been associated with significant virological or clinical events, such as liver disease progression, or HBeAg seroconversion [24,25], it is likely that these individuals harbour preC/C mutants that should be further investigated.

The limitation of this study was the lack of information on the treatment status of HBV-infected patients, which limits the robustness of the study.

## Conclusion

Our study unveiled that a high proportion of HBV-infected patients referred at *CPC* have normal values of biological parameters and thus are at a mild stage of disease progression. These results indicate that a small proportion of HBV-infected patients referred at *CPC* may ultimately be eligible for treatment according to the EASL classification. However, the presence of negative HBeAg serology in patients with high HBV VL levels also would suggest the presence of HBV pre-C mutant in this population that requires further investigation.

### What is known about this topic

- The Western Pacific Region and Africa are home to the majority of infected people;
- Among people with a chronic infection, between 20 and 30% could develop complications;
- Importance of treatment to avoid complications.

### What this study adds

- This study highlights the importance of monitoring biological markers;
- It also classifies and identifies patients eligible for treatment at the appropriate time.
